# Molecular mechanisms and multi-omics approaches in plant secondary metabolism: regulation, stress responses, and biotechnological applications

**DOI:** 10.3389/fpls.2026.1918176

**Published:** 2026-07-07

**Authors:** Muhammad Junaid Rao, Muhammad Tahir Ul Qamar, Bingsong Zheng

**Affiliations:** 1 State Key Laboratory for Development and Utilization of Forest Food Resources, Zhejiang A&F University, Hangzhou, China; 2 Zhejiang Key Laboratory of Non-wood Forest Products Quality Regulation and Processing Utilization, Zhejiang A&F University, Hangzhou, China; 3 Integrative Omics and Molecular Modeling Laboratory, Department of Bioinformatics and Biotechnology, Government College University Faisalabad (GCUF), Faisalabad, Pakistan

**Keywords:** abiotic and biotic stress, antioxidants, gene regulation and expression, molecular mechanisms, secondary metabolite (SM)

Plant secondary metabolites are not merely chemical curiosities; they are central mediators of ecological interactions, stress resilience, and human health. The staggering chemo-diversity encompassing alkaloids, flavonoids, terpenoids, and phenolic compounds underpins the medicinal, nutraceutical, and agronomic value of countless plant species. Yet, understanding the precise molecular wiring that governs *when*, *where*, and *how* these compounds are synthesized and accumulated has long been a formidable challenge. The advent of multi-omics technologies including integrating genomics, transcriptomics, metabolomics, and epigenomics has revolutionized this field, providing unprecedented resolution into the regulatory networks and biosynthetic highways of secondary metabolism. This Research Topic assembles 17 original contributions that collectively leverage these cutting-edge approaches to illuminate the tissue-specific dynamics, stress-responsive reprogramming, epigenetic control, and biotechnological potential of plant secondary metabolites. Far from a mere catalog of studies, the articles presented here converge on several transformative themes that are reshaping our fundamental understanding and practical exploitation of phytochemical diversity.

## Spatiotemporal dynamics and tissue-specific metabolic wiring

A recurring insight across multiple papers is that secondary metabolism is exquisitely compartmentalized across organs, tissues, and developmental stages. This spatial and temporal control is not a passive byproduct but an actively regulated process with profound implications for medicinal plant usage and molecular breeding. The integrated metabolomic and transcriptomic dissection of fenugreek (Dong et al.) pinpointed roots as the biosynthetic epicentre for key alkaloids, flavonoids, and terpenoids, and identified CYP450s, chalcone synthase, and MYB/bHLH transcription factors as the likely orchestrators of this tissue-specificity. Similarly, the work on *Bidens alba* (Wang et al.) demonstrated that flavonoids predominate in aerial tissues whereas certain sesquiterpenes and triterpenes accumulate in roots, with transcription factors such as BpMYB2 and BpbHLH1 showing contrasting expression between flowers and leaves. The developmental dimension was elegantly captured in *Cibotium barometz* (Wang et al.), where the mature rhizome—the traditional medicinal part—exhibited a pronounced enrichment of flavonoids driven by the upregulation of core phenylpropanoid pathway genes, providing molecular justification for optimized harvest timing. This principle extends to *Trichosanthes kirilowii* (Gao et al.), whose roots, fruits, pericarp, and seeds each harbor distinct bioactive profiles; the study not only mapped the tissue-biased accumulation of antitumor cucurbitacin B in roots but also identified Tk_ERF4 as a hub transcription factor linking terpenoid and flavonoid biosynthesis. Taking spatial resolution a step further, the spatial metabolomics investigation of areca inflorescence tissues (Wen et al.) used mass spectrometry imaging to visualize alkaloid enrichment in ovule tissues and flavonoid localization in vascular bundle sheaths at near-cellular resolution, a feat that would have been unthinkable a decade ago. Beyond the species represented in this Topic, recent integrated metabolomic and transcriptomic surveys have extended these principles to other fruit crops, linking distinct MYB-driven phenolic profiles to variety identity in strawberry (Rao et al., 2026) and kiwifruit (Duan et al., 2026), underscoring the conserved regulatory logic across diverse taxa.

Even post-harvest processing, often overlooked in molecular studies, reshapes tissue-level metabolite landscapes. The loquat flower metabolomics study (Duan et al.) demonstrated that freeze-drying best preserves terpenoid diversity and antioxidant capacity, whereas hot-water extraction—a common step—causes a dramatic 59–70% reduction in terpenoid content, directly informing nutraceutical processing pipelines. Collectively, these papers establish that the “where” and “when” of biosynthesis are as critical as the “what”, and that multi-omics is the tool of choice to decode these spatiotemporal signatures for precision agriculture and phytopharmacy.

## Stress-responsive metabolic reprogramming and protective mechanisms

Plants dynamically reconfigure their secondary metabolism in response to abiotic and biotic challenges, and this Research Topic showcases how multi-omics approaches disentangle these complex responses. High-temperature stress, a pressing climate resilience concern, was tackled in *Rhododendron moulmainense* (Khan et al.). The study revealed that severe heat (42 °C) up-regulates genes and metabolites in starch-sucrose metabolism and ABC transporter pathways, likely fueling osmoprotectant synthesis and secondary metabolite-mediated detoxification to sustain growth under thermal duress. In a complementary abiotic stress context, the genome-wide characterization of voltage-dependent anion channels (VDACs) in upland cotton (Akram et al.) identified 18 GhVDAC genes, of which GhVDAC6, 11, 13, and 15 were validated as responsive to drought and salt, laying a foundation for engineering mitochondrial transport to modulate stress-associated metabolite fluxes.

Biotic stress management also benefits from mechanistic insight. The field study on bacterial blight of pomegranate (Mustafa et al.) demonstrated that a copper-streptocycline combination not only suppressed disease but also enhanced fruit antioxidant capacity and secondary metabolite-linked quality traits, revealing a tangible link between chemical intervention and induced metabolic resilience. Another dimension of stress-associated metabolic modulation is seen in the maize kernel hardness study (Zhou et al.), where the interplay between starch-sucrose metabolism and carotenoid accumulation was implicated in the formation of a dense, vitreous endosperm, a trait of enormous economic importance for grain quality and post-harvest stability. The authors propose a model in which reduced sucrose synthase activity favors a compact polygonal starch architecture while carotenoids reinforce cellular interfaces, a vivid example of primary-specialized metabolic crosstalk in shaping stress-tolerant phenotypes. Such crosstalk is not limited to maize; engineering carbon partitioning has emerged as a promising strategy to enhance crop resilience to heat and other stresses, with recent work demonstrating that precise manipulation of carbon flux can bolster thermotolerance while maintaining yield (Zhao et al., 2025).

Phytohormonal gateways to secondary metabolism are further illuminated by the brassinolide (BL) application study in American ginseng (Zhao et al.). BL treatment at low concentration boosted growth and rare ginsenoside accumulation up to 21-fold, whereas high concentration suppressed growth but amplified rare ginsenosides up to 75-fold, pinpointing β-amyrin synthase and specific CYP450s as critical control nodes. This dose-dependent yin-yang between growth and defense metabolisms provides a tangible strategy for biotechnological yield enhancement. Similarly, exogenous methyl jasmonate has been shown to synergistically enhance phenolic compound accumulation and reactive oxygen species scavenging, thereby alleviating drought-induced oxidative damage in *Ilex rotunda* (Guo et al., 2026), highlighting the conserved role of hormonal elicitors in stress-primed phytochemical fortification. These examples reinforce the broader principle that transcription factors serve as central hubs that integrate stress signals and metabolic output.

## Epigenetic and light-mediated regulation of secondary metabolism

The regulatory layers above the genome are increasingly recognized as powerful levers of metabolic output. One of the most striking contributions of this Research Topic is the demonstration that chromatin accessibility directly governs secondary metabolite pathways. In hemp inflorescences (Ma et al.), the integration of ATAC-seq, transcriptomics, and metabolomics revealed that open chromatin at flavonoid biosynthetic gene promoters directly boosted flavonoid accumulation, while differential accessibility of fatty acid biosynthesis and trichome-identity genes indirectly shaped cannabinoid profiles between cultivars. This study moves us beyond static transcript factor-binding models into a dynamic chromatin landscape that can be harnessed for precision breeding and epigenetic engineering. The role of epigenetics in stress adaptation is a rapidly expanding field, and complementary findings in other crops have shown that histone modifications and chromatin remodeling can orchestrate genome-wide responses to environmental challenges, offering additional layers for metabolic engineering (Delarue et al., 2024).

Light quality, a perennial environmental variable, exerts equally profound effects. The investigation of blue polarized light on *Dendrobium officinale* (Li et al.) showed that blue polarized light, in contrast to ordinary blue or white light, triggered reddish stem coloration and significantly enriched flavonoid and phenylpropanoid metabolites, accompanied by distinct transcriptomic shifts in plant hormone signal transduction and zeatin biosynthesis. The identification of specific marker metabolites like melanoside A and rhapontigenin 3’-O-glucoside provides actionable targets for optimizing light regimes in controlled-environment cultivation of high-value medicinal orchids.

## Gene family evolution and functional genomics in metabolic pathways

Understanding the genomic architecture of enzyme families that synthesize, transport, or modify secondary metabolites is essential for rational pathway engineering. Beyond the stress-focused VDAC work mentioned earlier, this collection features a comprehensive analysis of the cellulose synthase (CESA) gene family in autopolyploid sugarcane (Wei et al.). While cellulose is a primary metabolite, its synthesis intersects profoundly with secondary metabolism and overall plant architecture. The identification of 30 SsCESA genes, their segmental duplication-driven expansion, and the experimental validation of SsNST1 binding to promoters of SsCESA4/7/9 illustrate how primary metabolic gene families can be mined for stress-resilient biomass improvement, indirectly affecting the carbon flux available for secondary metabolite production in this critical bioenergy crop. Such foundational genomic resources are indispensable for future multi-omics syntheses.

## Methodological advances and safe valorization of plant resources

A unified analytical platform remains a bottleneck in multi-omics studies, and this Topic advances the field with a standardized LC-MS/MS method for phytohormone profiling across five diverse plant matrices (Hakeem et al.). By marrying matrix-specific extractions with a common chromatographic and mass spectrometric setup, the authors obtained robust, cross-species phytohormonal signatures, revealing how cardamom’s high SA and ABA levels mirror its arid-adaptation strategy while aloe vera’s drought tolerance is reflected in lower hormone titers. Such methodological harmonization is a prerequisite for comparative meta-analyses and translational research. Equally important is the comparative metabolomics of kiwifruit varieties (Duan et al.), which profiled 309 terpenoids across five cultivars and highlighted the enrichment of ursane-type triterpenes and potent antioxidant capacity in *A. arguta* ‘Danyang’, offering a metabolic roadmap for breeding nutrient-dense fruit.

Finally, a particularly compelling contribution addresses the safe consumption of a traditionally toxic plant through the lens of multi-omics (Gao et al.). This study dissected the Bai nationality’s practice of removing androecium and gynoecium before processing *Rhododendron decorum* flowers. Multi-omics revealed that potentially harmful flavonoids are highly enriched in the removed floral organs, while traditional processing down-regulates toxins like epigallocatechin and myricetin while retaining flavor compounds and up-regulating beneficial p-coumaroyl quinic acid and taxifolin. This work beautifully illustrates how indigenous knowledge, when validated by modern systems biology, can guide the safe, evidence-based valorization of underutilized plant resources, turning a toxic risk into a functional food.

## Synthesis and future perspectives

The collection of articles in this Research Topic attests to the transformative power of multi-omics approaches in plant secondary metabolism research. Several cross-cutting themes emerge. First, the integration of transcriptomics and metabolomics is now a *de facto* standard, enabling the identification of candidate genes, transcription factors, and regulatory modules that directly govern metabolic flux. Second, spatial and temporal resolution is advancing rapidly, from tissue-level dissection to *in situ* mass spectrometry imaging, promising a future where single-cell multi-omics will map metabolic pathways at unprecedented granularity. Third, epigenetic and environmental inputs, chromatin state, light quality, and hormone crosstalk are being decoded as master switches that can be tuned for desired phytochemical outputs. Fourth, the translational arc from molecular discovery to biotechnological application is shortening, with multiple studies providing concrete molecular targets for breeding stress-tolerant, nutrient-dense, and pharmacologically enhanced crops. Fifth, traditional knowledge systems are being harmonized with high-throughput science to validate and optimize sustainable use practices.

However, challenges remain. The sheer complexity of multi-omics datasets demands robust computational frameworks and community-accessible databases to move from correlation to causation. Functional validation of the many predicted regulators—such as the MYB, bHLH, and ERF transcription factors spotlighted across these articles—will require efficient genetic transformation or gene editing pipelines in non-model medicinal species. Furthermore, the dynamic interplay between primary and secondary metabolism, exemplified by the starch-sucrose-carotenoid nexus in maize and the growth-defense trade-off in ginseng, calls for a systems-level metabolic modeling approach that can predict metabolic outcomes under multifactorial scenarios. Promisingly, strategies such as precise carbon partitioning engineering (Zhao et al., 2025) and the manipulation of transcription factor hubs that coordinate stress responses are already pointing the way toward predictive metabolic design.

We are confident that this Research Topic not only captures the state-of-the-art but also catalyzes future investigations that will deepen our mechanistic understanding and unlock the full biotechnological potential of the plant kingdom’s chemical treasure. The road from gene to metabolite is being mapped with ever-greater clarity, and the contributions herein are milestones on that journey.
